# Ultrafast electron dynamics in a topological surface state observed in two-dimensional momentum space

**DOI:** 10.1038/s41598-023-32811-1

**Published:** 2023-04-09

**Authors:** J. Reimann, K. Sumida, M. Kakoki, K. A. Kokh, O. E. Tereshchenko, A. Kimura, J. Güdde, U. Höfer

**Affiliations:** 1 Fachbereich Physik und Zentrum für Materialwissenschaften, Philipps-Universität, 35032 Marburg, Germany; 2 Graduate School of Science, Hiroshima University, 1-3-1 Kagamiyama, Higashi-Hiroshima, 739-8526 Japan; 3 Materials Sciences Research Center, Japan Atomic Energy Agency, Sayo, Hyogo 679-5148 Japan; 4 International Institute for Sustainability with Knotted Chiral Meta Matter (SKCM2), 1-3-2 Kagamiyama, Higashi-Hiroshima, 739-8511 Japan; 5 V.S. Sobolev Institute of Geology and Mineralogy SB RAS, 630090 Novosibirsk, Russian Federation; 6 Rzhanov Institute of Semiconductor Physics SB RAS, 630090 Novosibirsk, Russian Federation

**Keywords:** Topological insulators, Surfaces, interfaces and thin films, Electronic properties and materials, Spintronics

## Abstract

We study ultrafast population dynamics in the topological surface state of Sb$_2$Te$_3$ in two-dimensional momentum space with time- and angle-resolved two-photon photoemission spectroscopy. Linearly polarized mid-infrared pump pulses are used to permit a direct optical excitation across the Dirac point. We show that this resonant excitation is strongly enhanced within the Dirac cone along three of the six ${\bar{\Gamma }}$–${\bar{M}}$ directions and results in a macroscopic photocurrent when the plane of incidence is aligned along a ${\bar{\Gamma }}$–${\bar{K}}$ direction. Our experimental approach makes it possible to disentangle the decay of transiently excited population and photocurent by elastic and inelastic electron scattering within the full Dirac cone in unprecedented detail. This is utilized to show that doping of Sb$_2$Te$_3$ by vanadium atoms strongly enhances inelastic electron scattering to lower energies, but only scarcely affects elastic scattering around the Dirac cone.

## Introduction

The most remarkable properties of the topological surface state (TSS) of three-dimensional (3D) topological insulators (TIs) are its Dirac-like, quasi-relativistic energy dispersion and its helical spin texture in momentum (*k*-)space^1,2^. The latter locks momentum and spin of electrons in the TSS and suggests that momentum scattering within the Dirac cone is strongly suppressed with even complete absence of direct backscattering^3^. This implies that surface currents are not only automatically spin-polarized, but also flow ballistically over large distances. Together with the robustness of the TSS against nonmagnetic pertubations due its topological protection, this makes these surface states very promising for use in ultrafast low-loss electronics and spintronics. It turned out, however, that not only the application of 3D TIs in real devices, but already the confirmation of these unique properties by transport measurements is typically impeded by the dominant role of bulk carriers which are induced by intrinsic electron- or hole-doping^4,5^ in combination with the rather small band gap of even the prototype 3D TIs such as the binary chalcogenides Bi$_2$Se$_3$, Bi$_2$Te$_3$, and Sb$_2$Te$_3$.

Direct evidence for long-lasting ballistic surface currents has been provided by ultrafast pump-probe experiments that combine surface current generation and its time-resolved detection by angle-resolved photoelectron spectroscopy (ARPES). The most direct approach resolves on a subcycle time scale the momentum distribution of Dirac fermions close to the Fermi level $E_F$ of Bi$_2$Te$_3$ as they are accelerated by the carrier wave of a THz pulse^6^. Its dynamics have been described by the semi-classical Boltzmann equation which accounts for inelastic scattering as well as for elastic momentum scattering. Both corresponding scattering times have been found to be longer than 1 ps which shows that THz-accelerated Dirac fermions may propagate coherently over several hundred nanometres before under-going scattering^6^. In a preceding work, we have shown that such long momentum scattering times are also inhere in electrons that are optically excited into the initially unoccupied upper branch of the Dirac cone in intrinsically p-doped Sb$_2$Te$_3$^7^. This has been accomplished in a time- and angle-resolved two-photon photoemission (2PPE) experiment by using linearly polarized mid-IR laser pump pulses. These pulses do not only permit a direct optical excitation between the occupied lower and initially unoccupied upper branch of the TSS, but also generate a strong population asymmetry along a given direction in *k*-space parallel to the surface which is enhanced at an energy of a few hundred meV above $E_F$^7^. In both experiments, the momentum distribution of the electrons in the TSS has been recorded in one direction of the two-dimensional (2D) momentum space of the surface and the current has been deduced from its asymmetry for opposite parallel momenta along the direction of current flow. The extracted momentum scattering times therefore represent effective phenomenological times for backscattering (180$^\circ$ scattering), although multiple scattering processes with smaller scattering angles are in fact involved, because direct backscattering should be completely suppressed in the TSS^8^.

Here, we present the investigation of the ultrafast population dynamics in the TSS of Sb$_2$Te$_3$ after direct optical excitation by mid-IR pulses in the full 2D momentum space. We will show that the excitation is strongly enhanced along three of the six ${\bar{\Gamma }}$–${\bar{M}}$ directions, which allows us to monitor the subsequent redistribution of the electrons within the whole Dirac cone in unprecedented detail. We find that the enhancement significantly differs along these three directions, if a mirror plane (${\bar{\Gamma }}$–${\bar{M}}$ direction) of the sample surface is oriented perpendicular to the plane of light incidence. This confirms that the excitation by linearly polarized mid-IR pulses can in fact generate a macroscopic photocurrent which is automatically spin polarized due to the spin texture of the TSS.

The time-resolved observation of the decay and the redistribution of the initially inhomogeneous TSS population in 2D momentum space allows to disentangle inelastic decay out of the TSS and elastic momentum scattering within the TSS in great detail. We demonstrate this capability for pristine and vanadium doped Sb$_2$Te$_3$ and show how the vanadium atoms affect these two scattering processes in a distinctly different way. The dominating mechanism for inelastic decay in p-doped samples such as intrinsicially p-doped stoichiometric Sb$_2$Te$_3$ has been identified by conventional 2PPE experiments to be electron-hole pair creation in the partially filled valence band^9–11^. The corresponding population lifetime therefore strongly depends on the position of $E_F$ and it could be shown that it can be strongly enhanced by Fermi level tuning through doping^12,13^. Consequently, the emergence of additional states in the bulk band gap can be expected to introduce further decay channels and we have in fact shown that doping of Sb$_2$Te$_3$ by vanadium strongly reduces the population lifetime even for small concentrations of a few percent. This has been attributed to V-induced impurity states which have been spectroscopically identified by scanning tunneling spectroscopy (STS)^14,15^. We show here that the momentum scattering is on the other hand surprisingly almost unaffected by the presence of vanadium atoms, which demonstrates the robustness of momentum scattering in the TSS against defects even for magnetic scattering centers.

## Experimental method

Details of the optical setup are described in Refs.^7,14^. Electrons were excited above the Fermi level $E_F$ by pump laser pulses with a photon energy in the mid-IR ($h\nu _{\textrm{pump}}=0.33$ eV, 100 fs) or in the visible (VIS) ($h\nu _{\textrm{pump}}=2.52$ eV, 80 fs) and subsequently photoemitted by time-delayed ultraviolet (UV) probe laser pulses ($h\nu _{\textrm{probe}}=5.04$ eV, 100 fs) at a repetition rate of 200 kHz. The chosen UV photon energy just suppresses direct photoemission of the occupied states but provides full access of the unoccupied part of the TSS in 2PPE with high dynamic range. Pump and probe pulses were *p*-polarized and focused on the sample into a spot with a diameter of $\sim 100$ $\upmu$m at angles of incidence of both beams close to 45$^\circ$. The experiments were carried out in a $\mu$-metal shielded UHV chamber at a base pressure of $4\times 10^{-11}$ mbar with the samples cooled to 110 K after in-situ cleaving by the Scotch tape method. Characterization of the samples, which were grown by the modified vertical Bridgman method with a rotating heat field^16^, is described in detail in Ref.^14^. Photoelectrons were collected by a hemispherical analyzer (Scienta DA30) equipped with deflection plates in the electron lens which makes it possible to aquire energy-momentum (*E*–$k_x$) maps with the electron momentum $k_x$ oriented along the entrance slit of the hemisphere for varying momentum $k_y$ (perpendicular to $k_x$). In this way, the ultrafast dynamics of the optically excited population in the initially unoccupied band structure can be sequentially mapped in the full two-dimensional momentum space of the sample surface without moving the sample.

## Results and discussion

### Homogenous population of the TSS by visible excitation

Before turning to the discussion of the dynamics of the momentum distribution of photocurrents excited by mid-IR pump pulses, we first discuss data taken with visible pump pulses in order to disentangle the impact of the pump and probe pulses on the energy and momentum distribution of the detected final states. The latter is governed by both the transient population of the intermediate state excited by the pump pulses and the sequential photoemission by the probe pulses into the detected final states. Visible pump pulses have been shown to initially excite electrons far above the TSS and result in an indirect population of the Dirac cone^9,17^. This involves many scattering events which completely homogenize the electron distribution in momentum space^7^. Therefore, any momentum dependence of the spectral weight of the 2PPE data can be in this case related to the photoemission probe process and can be used to correct the momentum-dependent 2PPE data for the optical matrix element of the probe process as we have shown in Ref.^18^.

**Figure 1:**
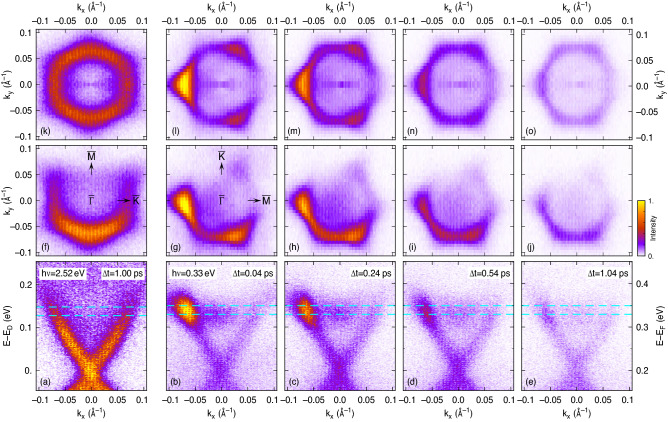
Angle-resolved 2PPE data of Sb$_2$Te$_3$ excited by visible ($h\nu =2.52$ eV) and mid-IR ($h\nu =0.33$ eV) pump pulses. (**a**) *E*–$k_x$ map for 2.52-eV excitation and $k_x$ along ${\bar{\Gamma }}$–${\bar{K}}$ at a pump-probe delay of $\Delta t=1$ ps. (**b**–**e**) For 0.33-eV excitation and $k_x$ along ${\bar{\Gamma }}$–${\bar{K}}$ at different pump-probe delays as indicated. (**f**–**j**) Corresponding $k_x$–$k_y$ maps integrated over energy intervals centered at $E-E_D=140$ meV as is depicted by the cyan dashed lines in (**a**–**e**). (**k**–**o**) The same cuts as (**f**–**j**) but with the intensity corrected for the matrix element of the probe transition and symmetrized by mirroring the data at the $k_x$-axis. For all data, the plane of light incidence is oriented along the $k_y$-axis.

Figure 1a,f show two selected cuts through 2PPE data of the initially unoccupied part of the TSS recorded at 1 ps after excitation with visibile pump pulses of photon energy $h\nu =2.52$ eV. At this delay, the indirect population of the TSS has reached its maximum^9^. The *E*–$k_x$ map for $k_y=0$ depicted in Fig. 1a is symmetric in $k_x$ and shows the linear dispersion of the TSS along ${\bar{\Gamma }}$–${\bar{K}}$ with the Dirac point (DP) located 200 meV above $E_F$. The TSS is homogeneously populated in energy up to the bulk conduction band minimum at $E-E_F\approx 330$ meV which feeds the population of the TSS for several ps^9^. The $k_x$–$k_y$ map centered at $E-E_D=140$ meV depicted in Fig. 1f shows the expected warping of the Dirac cone at this energy with a slight flattening of the linear dispersion along ${\bar{\Gamma }}$–${\bar{M}}$^19^, but most notably a strong asymmetry of the 2PPE data with respect to $k_y$ with a half-moon shaped intensity distribution. This asymmetry does not result from an inhomogeneous population of the TSS, but from the oblique incidendence of the p-polarized UV probe pulses. This momentum distribution is independent of the sample orientation as has been tested by azimuthal rotation of the sample and measurements with two different orientations of the plane-of-incidence. For *p*-polarized probe pulses incident along the $k_y$-direction as presented in Fig. 1, a half moon shaped intensity distribution indicates that the TSS is dominated by out-of-plane $sp_z$ orbitals with neglible in-plane contributions which would show a threefold symmetric pattern^20^. The $k_x$–$k_y$ maps can be therefore corrected for the photoemission probe process by dividing the intensity by $(1-\sin \phi )$ where $\phi$ is the azimuthal angle counting anticlockwise with respect to the $+k_x$ direction. This is, however, not applicable for $\phi$ close to 90$^\circ$ and we apply this correction only for $k_y<0$ and mirror the data with respect to the $k_x$ axis. The corrected and symmetrized data depicted in Fig. 1k shows a homogeneous intensity distribution around the Dirac cone and in particular no kink at the mirror axis which demonstrates that this correction accounts well for the matrix element of the UV probe pulses. This makes it now possible to correct the 2PPE data to reveal the actual population in the intermediate state also for other pump photon energies, as long as the same photon energy and polarization of the photemission probe is kept. Mirroring of the data, however, is applicable only when either the population is homogeneous in *k*-space or if the plane of probe incidence is oriented perpendicular to a mirror axis of the sample surface such as the ${\bar{\Gamma }}$–${\bar{M}}$ direction of the threefold symmetric surface of Sb$_2$Te$_3$(0001).

### Photocurrent generation by mid-IR excitation

The four rightmost columns of panels in Fig. 1 show 2PPE data for mid-IR pump pulses which drive a resonant excitation across the Dirac point^7^ and result in a strongly enhanced population centered at $E-E_D=140$ meV as can be most clearly seen in Fig. 1b. Moreover, the mid-IR excitation induces a strong asymmetry with respect to $k_x$ when the sample is oriented with the ${\bar{\Gamma }}$–${\bar{M}}$ direction aligned perpendicular to the plane of probe incidence. As can be already seen in the raw and uncorrected $k_x$–$k_y$ map (Fig. 1g), the population is not only enhanced in one direction, but in three of the six ${\bar{\Gamma }}$–${\bar{M}}$ directions. Even though the photoemission probe is much less efficient in the direction of the upper right ${\bar{M}}$ point, the enhancement is still faintly visible in the uncorrected data. The threefold pattern indicates that the optical excitation is associated with the Sb–Te bonds which have a threefold arrangement in the unit cell^21^. The degree of the population enhancement, however, differs in the three directions. It is much stronger in direction of the left as compared to that of the lower right ${\bar{M}}$ point. This difference is even further enhanced when the data is corrected for the photoemission probe as shown in Fig. 1l. This clearly demonstrates that the direct excitation by the mid-IR pulses in fact generates a macroscopic photocurrent along the $k_x$ direction while an asymmetry in a *E*–$k_x$ cut as shown Fig. 1b could also result from a threefold symmetric excitation with equal weight along the ${\bar{\Gamma }}$–${\bar{M}}$ directions as noticed in Ref.^22^. The generation of a macroscopic current in the ${\bar{\Gamma }}$–${\bar{M}}$ direction by p-polarized light incident along the ${\bar{\Gamma }}$–${\bar{K}}$ direction is in agreement with the observation on linearly polarized THz emission in Bi$_2$Te$_3$ by an p-polarized optical pump for the same geometry, which has been attributed to the generation of a nonlinear current^23^. Also the decomposition of helicity-dependent THz emission spectroscopy^24^ and experiments that measure photocurrents by electrical contacts^25^ into different contributions of surface and bulk currents draw conclusions on surface currents that reflect the three-fold symmetry of the (111) TI surfaces. In these experiments, however, it is difficult to unambiguously identify the specific contribution of the intrinsic TSS at $E_F$ because also higher-lying topological protected surface states might be involved, in particular when pump light in the visible or near infrared is used^26^. In contrast, time- and angle-resolved photoelectron spectroscopy provides a most direct approach for the observation of currents in a specific electronic state. Even when no macroscopic current is generated, this method makes it possible to gain information about elastic momentum scattering of the electrons within the Dirac cone by the investigation of the population balancing along a line in *k*-space that shows a population asymmetry^7,27^.

### Dynamics of momentum scattering

The time-resolved observation of the redistribution in the full two-dimensional *k*-space of the surface studied here, however, provides a much more detailed insight into the initial excitation and the sequential scattering processes. This is demonstrated by the data of Fig. 1b–e, g–j and k–o which shows a time series of cuts through the 2PPE data for selected delays $\Delta t$ between mid-IR pump and UV probe. These data show that the population at the resonant excitation energy of $E-E_D=140$ meV has almost reached its maximum already at $\Delta t=40$ fs because it is created by a direct optical transition from the lower to the upper part of the Dirac cone^7^. For increasing delay, the whole population at the resonant excitation energy gradually decreases due to inelastic scattering of the electrons. Simultaneously, the population around the Dirac cone becomes more uniform as can be seen in the $k_x$–$k_y$ maps. Electrons are elastically scattered in direction within the cone that were initially less populated. However, even at $\Delta t = 1.04$ ps, an enhancement of the population along the ${\bar{\Gamma }}$–${\bar{M}}$ directions is still clearly visible that shows that elastic scattering, which randomize the population around the Dirac cone, is weak as compared to inelastic scattering. This results from the suppression of momentum scattering due to the helical spin structure of the TSS and is in strong contrast to the electron dynamics in topological trivial materials such as GaAs where the electrons in the $\Gamma$ valley have been shown to quasiequilibrate in momentum space within 100 fs^28^. The *E*–$k_x$ maps show that the filling of the lower part of the Dirac cone is delayed with respect to the population at the resonant excitation energy. This is caused by sequential inelastic intraband scattering of the directly excited electrons to lower energies. The intensity at all energies below the resonant excitation energy still increases from $\Delta t=0.04$ ps to $\Delta t=0.24$ ps and decays only for longer delays whereas the resonantly populated part at negative $k_x$ is already reduced at $\Delta t=0.24$ ps. This is a similar time scale as has been recently observed for the inelastic intraband dynamics on bulk insulating Bi$_{1.4}$Sb$_{0.6}$Te$_{1.51}$Se$_{1.49}$ (BSTS) by THz-induced transient THz reflectivity measurements^29^.

**Figure 2:**
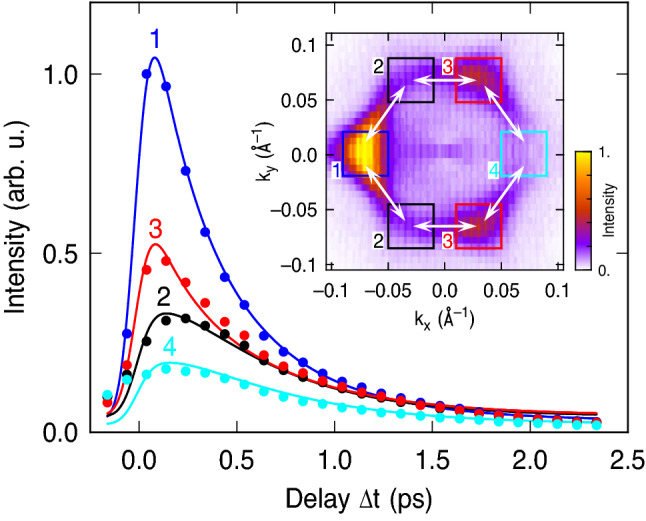
Time-evolution of the 2PPE intensity at different points of the $k_x$–$k_y$ maps (c.f. Fig. 1l–o) as is indicated by the square integration areas in the inset (data points). The solid lines show fits of a rate-equation model for the population of the Dirac cone at these points considering inelastic electron scattering to lower energies and elastic scattering between adjacent areas as indicated by the white arrows in the inset.

The redistribution of the electrons due to elastic scattering within the TSS is analyzed in more detail in Fig. 2, where we present the time-evolution of the 2PPE intensity at the resonantly excited energy along the different ${\bar{\Gamma }}$–${\bar{M}}$ directions. For this purpose, the 2PPE intensity, which is proportional to the TSS population, has been integrated over four square regions shown in the inset of Fig. 2. The data show that the populations in these regions all decay after their differently strong initial excitation, but equalize at later delays. At first, the populations in regions 2 and 3 equalize at $\Delta t > 0.5$ ps followed by equalizing with the initially largest population in region 1 at $\Delta t > 1$ ps. The population in region 4 also approaches the others, but even at the largest observed delay of 2.3 ps it is still the smallest. The alignment of the populations shows that the decay of the population in the different regions is not independent and not only governed by inelastic scattering, but also accompied by exchange between the regions due to elastic momentum scattering. The latter can be characterized by a weighted distribution of scattering angles whereas the spin structure of the TSS suggests an enhanced probability for small angle scattering and a complete suppression of direct backscattering^8^.

We analyze our data by a simplified rate-equation model for the transient populations $n_i$ in the four regions $i=1\ldots 4$ by considering population loss of all regions with a common rate $\Gamma ^d=1/\tau ^d$ and population exchange between neighboring regions with a common rate $\Gamma ^e_{60^\circ }=1/\tau ^e_{60^\circ }$ as indicated by the white arrows in the inset of Fig. 2. Here, $\tau ^d$ and $\tau ^e_{60^\circ }$ are the corresponding mean scattering times for inelastic scattering to lower energies within the TSS or into the bulk and for $60^\circ$ momentum scattering, respectively. Considering that the regions 2 and 3 centered at $k_y~-0.06$ Å$^{-1}$ in the inset of Fig. 2 are identical to those centered at $k_y~+0.06$ Å$^{-1}$ due to the mirroring of the data, the rate equations are given by

1 $$\begin{aligned} \frac{dn_1}{dt}= & {} A_1{\delta }(t)-{\Gamma ^{d}}{n_{1}}+{\Gamma ^{e}_{60^\circ }}(2{n_{2}}-2{n_{1}}),\nonumber \\ \frac{dn_2}{dt}= & {} A_2{\delta }(t)-{\Gamma ^{d}}{n_{2}}+{\Gamma ^{e}_{60^\circ }}({n_{1}}+{n_{3}}-2{n_{2}}),\nonumber \\ \frac{dn_3}{dt}= & {} A_3{\delta }(t)-{\Gamma ^{d}}{n_{3}}+{\Gamma ^{e}_{60^\circ }}({n_{2}}+{n_{4}}-2{n_{3}}),\nonumber \\ \frac{dn_4}{dt}= & {} A_4{\delta }(t)-{\Gamma ^{d}}{n_{4}}+{\Gamma ^{e}_{60^\circ }}(2{n_{3}}-2{n_{4}}). \end{aligned}$$

Here ${\delta }(t)$ is the temporal intensity profile of the Gaussian shaped mid-IR laser pulse, and the $A_i$ indicate the different excitation probabilities in the four different regions. This model represents an extension of the rate-equation model used in Ref.^7^ where elastic scattering was characterized by an effective scattering time for 180$^\circ$ scattering which is in fact composed of sequential scattering events with smaller scattering angles. The solid lines in Fig. 2 show the best fit of the data within our model assuming that the 2PPE intensity is proportional to the TSS population. This fit yields an elastic scattering time of $\tau ^e_{60^\circ }=1.21(15)$ ps for 60$^\circ$ scattering and an inelastic scattering time of $\tau ^d=0.44(10)$ps. The number for $\tau ^e_{60^\circ }$ roughly fits to the previously determined effective scattering time of 2.5 ps for 180$^\circ$ scattering^7^. The latter would correspond to a population exchange only between regions 1 an 4. The inelastic scattering time is about 30% smaller as reported in Ref.^7^, but within the variation range observed for different cleaves of the sample, which has been attributed to variations of the defect density and of the position of $E_F$ with respect to $E_D$ and the valence band maximum^9^.

### Impact of V-doping

In the following, we will show that the capability to observe the electron dynamics in the full two-dimensional momentum space makes it possible to disentangle elastic and inelastic electron scattering, even if no macroscopic photocurrent is generated, as long as the initial excitation is inhomogeneous in momentum space. For this purpose, we compare the electron dynamics of pristine and vanadium doped Sb$_2$Te$_3$ for a different sample orientation. Recently, we have shown that even small vanadium concentrations of a few percent drastically reduce the inelastic scattering time in Sb$_2$Te$_3$, which was attributed to impurity states^14,15^. This has been deduced from the observation of the dynamics after mid-IR excitation in one-dimensional cuts through the TSS band structure along one surface direction.

**Figure 3:**
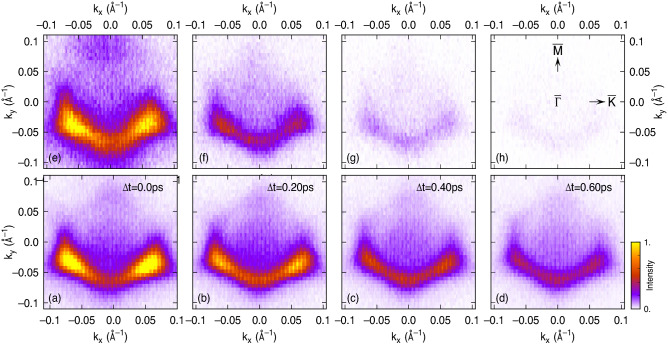
Comparison of the population dynamics between pristine Sb$_2$Te$_3$ (**a**–**d**) and Sb$_{2-x}V_x$Te$_3$ with $x=0.015$ (**e**–**h**). The panels show uncorrected $k_x$–$k_y$ maps centered at $E-E_D=141$ meV for different time delays $\Delta t$. The sample orientation is indicated in panel (**h**). The plane of light incidence is along the $k_y$-direction.

**Figure 4:**
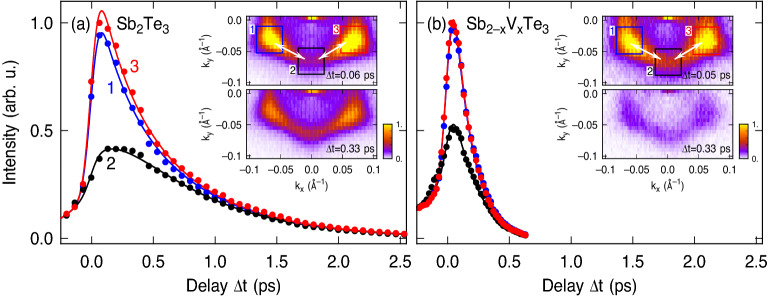
Comparison of the population dynamics between pristine (**a**) Sb$_2$Te$_3$ and (**b**) Sb$_{2-x}$V$_x$Te$_3$ with $x=0.015$ with the ${\bar{\Gamma }}$–${\bar{M}}$ direction aligned along the plane of incidence. The insets show $k_x$–$k_y$ maps centered at $E-E_D=140$ meV for two selected time delays $\Delta t$. Data points show the intensity integrated over the blue and red square areas depicted in the $k_x$–$k_y$ maps. Solid lines show fits to the data.

In Fig. 3, we present here a comparison of time-resolved 2PPE data in the two-dimensional momentum space obtained for pristine Sb$_2$Te$_3$ and a Sb$_{2-x}$V$_x$Te$_3$ sample with $x=0.015$. The measurements were done at a temperature of 110 K, which is well below the Debye temperature of Sb$_2$Te$_3$ ($\Theta _D = 162$ K^30^), but at the same time well above the Curie temperature of V-doped Sb$_2$Te$_3$^14^. Both samples were equally oriented with the ${\bar{\Gamma }}$–${\bar{M}}$ direction aligned along the plane of incidence. This results in a pure threefold symmetric pattern of the excited population in the TSS and no macroscopic photocurrent is generated, as can be most clearly seen in Fig. 3a,e for both samples, respectively. Information about elastic momentum scattering, however, can still be extracted from these data because of the initially strong selective enhancement of the excited population in three of the ${\bar{\Gamma }}$–${\bar{M}}$ whereby only the lower two are clearly observed in the raw data because of the matrix element of the probe transition. This is demonstrated in Fig. 4 where we show an analysis of the transient TSS population at the resonantly excited energy for both data after correction of the 2PPE intensity for the probe matrix element. Symmetrization by mirroring is not possible for this threefold symmetric data because the ${\bar{\Gamma }}$–${\bar{K}}$ direction is not a mirror plane. Similar to the analysis shown in Fig. 2, the 2PPE intensity has been evaluated at the three visible ${\bar{M}}$-points. The blue and red solid dots show these data for two of the initially differently populated ${\bar{M}}$-points as indicated by the blue and red rectangles in the $k_x$–$k_y$ map of the inset, respectively. The solid lines show again fits of the corresponding rate-equation model considering inelastic decay and 60$^\circ$ elastic momentum scattering. In order to well describe the initial rise of the transients, we additionally consider a small contribution of the third image-potential state to the 2PPE intensity, which is only visible at negative delays (not shown). It appears at similar final state energies as the TSS but with pump and probe pulses exchanged^18,27^. Its rise and decay therefore appears towards negative delays here.

From the data of the pristine sample shown in Fig.4a, we obtain in this way an inelastic decay time of $\tau ^d=0.68(1)$ps which is very close to the result of Ref.^7^, but longer by $\sim 50\%$ if compared to the one determined from the data in Fig. 2 due to the variations for different cleaves as discussed above. In contrast, the elastic momentum scattering time $\tau ^e_{60^\circ }=1.40(5)$ is comparable to the one obtained from the data of Fig. 2 which already indicates that elastic scattering is rather robust against defects and coupling to the bulk. Comparing Fig. 4a and b makes it apparent that V-doping strongly reduce the inelastic scattering time, and we determine a four times smaller value of $\tau ^d=0.16(1)$ps as compared to pristine Sb$_2$Te$_3$. This can be most probably attributed to the V-induced impurity states that have been identified by scanning tunneling spectroscopy (STS)^14^. Such impurity states can introduce additional decay channels for intraband scattering to lower energies and were shown to persist even in the presence of magnetic order^31^. Surprisingly, however, the momentum scattering time is not much affected by V-doping as can be already seen by comparing the 2D maps of the pristine and V-doped sample in the insets of Fig. 4 taken at 0.33 ps. Although the overall intensity for the V-doped sample is more strongly reduced as compared to the pristine sample due to the stronger inelastic scattering, the inhomogeneity of the intensity distribution around the Dirac cone is comparable for both samples. This can be even more clearly seen by comparing the time dependence of the intensity in region 2 with those of region 1 and 3 shown in Fig. 4b. It does not only take considerable time for these intensities to align. The still long momentum-scattering time is also reflected by the fact that the maximum difference of these intensities, which is reached around 0.12 ps, is comparable to the one of the pristine sample. An accelerated momentum-scattering would lead to a faster alignment of these intensities. A fit of the data indeed gives only a slightly reduced momentum-scattering time of $\tau ^e_{60^\circ }=1.0(5)$ ps, but with an enhanced uncertainty due to the stronger inelastic scattering. Such still weak momentum scattering is surprising because it might be expected that impurities also enhance momentum scattering, in particular if the impurities carry a magnetic moment. On the other hand, time-resolved quantum-beat photoelectron spectroscopy of image-potential states on a Cu(001) surface has shown that single scattering centers can affect inelastic decay and momentum scattering in a very different way depending on the details of the scattering potential^32^. While CO adatoms mainly lead to the decay of the quantum beats between different image-potential states due to momentum scattering^33^, an even much smaller concentration of Cu adatoms has a stronger impact on the inelastic decay^34^. Ab initio calculations suggest that for scattering of electrons in a TSS with single magnetic impurities the non-magnetic part of the scattering potential with resonant scattering into defect states dominates^35^. This is in line with our observation that the magnetic impurities strongly reduce the inelastic scattering time, but have only a small impact on elastic momentum scattering.

## Conclusions

We have experimentally investigated the ultrafast population dynamics of electrons in the TSS of pristine and V-doped Sb$_2$Te$_3$ in two-dimensional momentum space after direct optical excitation by linearly polarized mid-IR pulses using time- and angle-resolved two-photon photoemission. We have shown that the population at the resonant excitation energy in the upper Dirac cone is not uniform in momentum space, but enhanced along three of the six ${\bar{\Gamma }}$–${\bar{M}}$ directions. If the plane of incidence is aligned along one of the ${\bar{\Gamma }}$–${\bar{K}}$ directions, this enhancement is not threefold symmetric and corresponds to a macroscopic photocurrent along a ${\bar{\Gamma }}$–${\bar{M}}$ direction. The inhomgeneous excitation, together with the detection in the full two-dimensional momentum space of the surface, makes it possible to observe the redistribution of the population around the Dirac cone by elastic momentum scattering and to lower energies by inelastic scattering. We have found that momentum scattering is much less efficient as compared to inelastic scattering with strong supression of large-angle scattering, as is expected from the helical spin structure of the TSS. V-doping has been shown to strongly enhance inelastic scattering, while momentum scattering is almost unaffected, although the dopants carry a magnetic moment. This is in agreement with ab initio calculations which suggest that the scattering potential of single magnetic impurities is dominated by its non-magnetic part.

## Data Availability

The datasets generated during and/or analysed during the current study are available from the corresponding author on reasonable request.
